# Fabrication of a Novel Highly Sensitive and Selective Immunosensor for Botulinum Neurotoxin Serotype A Based on an Effective Platform of Electrosynthesized Gold Nanodendrites/Chitosan Nanoparticles

**DOI:** 10.3390/s17051074

**Published:** 2017-05-09

**Authors:** Rahim Sorouri, Hasan Bagheri, Abbas Afkhami, Jafar Salimian

**Affiliations:** 1 Department of Microbiology, Faculty of Medicine, Baqiyatallah University of Medical Sciences, Tehran 1435116471, Iran; 2 Chemical Injuries Research Center, Baqiyatallah University of Medical Sciences, Tehran 1435116471, Iran; h.bagheri82@gmail.com (H.B.); jafar.salimian@gmail.com (J.S.); 3 Faculty of Chemistry, Bu-Ali Sina University, Hamedan 6517838695, Iran; abbas.afkhami@gmail.com

**Keywords:** label-free immunosensor, botulinum neurotoxin, biosensors, gold nanodendrites, chitosan nanoparticles, screen printed carbon electrode

## Abstract

In this work, a novel nanocomposite consisting of electrosynthesized gold nanodendrites and chitosan nanoparticles (AuNDs/CSNPs) has been prepared to fabricate an impedimetric immunosensor based on a screen printed carbon electrode (SPCE) for the rapid and sensitive immunoassay of botulinum neurotoxin A (BoNT/A). BoNT/A polyclonal antibody was immobilized on the nanocomposite-modified SPCE for the signal amplification. The structure of the prepared nanocomposite was investigated by transmission electron microscopy (TEM), scanning electron microscopy (SEM), X-ray diffraction (XRD), Fourier transform infrared (FTIR) spectroscopy, cyclic voltammetry (CV), and electrochemical impedance spectroscopy (EIS). The charge transfer resistance (R*_CT_*) changes were used to detect BoNT/A as the specific immuno-interactions at the immunosensor surface that efficiently limited the electron transfer of Fe(CN)_6_^3−/4−^ as a redox probe at pH = 7.4. A linear relationship was observed between the %∆*R_CT_* and the concentration logarithm of BoNT/A within the range of 0.2 to 230 pg·mL^−1^ with a detection limit (S/N = 3) of 0.15 pg·mL^−1^. The practical applicability of the proposed sensor was examined by evaluating the detection of BoNT/A in milk and serum samples with satisfactory recoveries. Therefore, the prepared immunosensor holds great promise for the fast, simple and sensitive detection of BoNT/A in various real samples.

## 1. Introduction

Botulinum neurotoxin (BoNT), the most toxic substance known to man, remains one of the highest priority biological threat agents [1]. It is one of the most potent substances known, with a concentration as low as 1 ng/kg is estimated to be fatal to humans. For this reason it is classified in category A among the select agents list published by the Centers for Disease Control and Prevention [2]. It is viewed as a potential bioterrorism agent because BoNT can be produced in gram quantities using relatively simple biochemical techniques [3]. Among all BoNT serotypes, BoNT/A represents one of the most important bioterrorism agents because of its extreme toxicity and easy production [4]. In addition, BoNT/A has recently been exploited and widely used to treat disorders such as hyperhidrosis, cervical dystonia, focal spasticity, hemifacial spasm, ophthalmological and otolaryngeal disorders, in addition to applications in cosmetic industry [5].

The gold-standard method for BoNT/A detection is the mouse bioassay (MBA or lethality test) [6,7], however, this method is extremely time-consuming and slow (a typical assay taking a minimum of 48–72 h for the confirmation) and involves injection of the potential toxin-containing sample into live mice followed by observation for signs of botulinic paralysis or death [8]. The use of large numbers of animals makes the assay expensive as well as posing ethical conflicts. Furthermore, the maximum error for the MBA is above 30% and can be as high as 60% between different testing facilities.

To address the mentioned problems, several methods have been reported for the determination of BoNT/A such as sandwich chemiluminescence enzyme immunoassay (ELISA) [5], capillary electrophoresis (CE) [2], surface plasmon resonance (SPR) [9], chemiluminescent [10], fluorescence [8], Endopep-mass spectrometry (MS) [11,12] and high performance liquid chromatography (HPLC) [13]. These methods are generally slow, expensive and labor intensive. In recent years, because of the simple instrumentation required and easy quantification, electrochemical immunoassays have become the predominant analytical techniques for the quantitative detection of various biomolecules [14,15].

The main methodology for the fabrication of an impedimetric immunosensor is the immobilization of antibodies onto the surface of the electrodes. This strategy avoids the labeling step which is vital in other electrochemical biosensor fabrication methods and greatly shortens the detection time. Impedimetric sensors measure impedance, including resistance and capacitance parameters that originate from the result of an interaction with a small amplitude voltage signal as a function of frequency [16]. Measurements of *R_CT_* in antigen-antibody interaction analysis are more significant than measurements of current or potential changes [17].

One of the key factors to increase the sensitivity of immunoassays is the provision of a sufficient number of binding sites for biomolecule immobilization, which can be achieved through the use of high surface area substrates with high conductivity [8]. Because of the unique optical, electrochemical, and mechanical properties, nanomaterials can effectively increase the biomolecule loading and conductivity, which further results in a detection sensitivity enhancement [14,18,19].

Gold-based nanomaterials have attracted great attention in various research fields. Among the variety of Au nanostructures, there is a great interest for the preparation of gold nanodendrites (AuNDs), because of their high surface area and conductivity, finding promising applications in biosensors, electronic devices, and fuel cells [20]. For gold nanostructures synthesis, different methods are applied, including seeding, templating, electrodeposition, and wet chemical methods. Among these methods, the electrodeposition method is especially attractive owing to its more facile and eco-friendly preparation process [21].

Chitosan (CS) is a kind of matrix for biomolecule immobilization with attractive properties such as high film-forming ability, high permeability characteristics toward water, high adhesion, and biocompatibility. These characteristics make it suitable for use in the immobilization of biomolecules [22]. However, its application in the development of electrochemical sensors is limited because of its non-conductive properties. The combination of nanomaterials having unique electrochemical properties with CS affords potential materials for biosensor fabrication.

A survey of the literature shows two studies in recent years describing the impedimetric determination of BONT/A [15,23]. Afkhami and coworkers reported an impedimetric determination of BoNT/A using a Au nanoparticles/graphene-chitosan composite [15]. With this method, a BoNT/A linearity range of 0.27–268 pg·mL^−1^ with the detection limit of 0.11 pg·mL^−1^ was achieved. The preparation of the proposed immunosensor was also a time-consuming process (requiring about 20 h). However, in our investigation, the prepared nanocomposite based on AuNDs/CSNPs has relatively similar figures of merit without the use of graphene as a component of the sensing layer and a relatively short preparation process (14 h).

We thus propose a novel immunosensor for the impedimetric determination of BoNT/A based on immobilization of antibodies on the surface of a screen printed carbon electrode (SPCE) modified by AuNDs/CSNPs. The results showed that the impedimetric signals of BoNT/A are significantly improved in the presence of AuND/CSNPs as an excellent substrate of sensing layer. Under optimized conditions, the results showed that the proposed immunosensor could be successfully employed for the determination of BoNT/A in spiked milk and serum samples.

## 2. Materials and Methods

### 2.1. Reagents and Apparatus

Chitosan (CS, 350,000 g·mol^−1^ and degree of deacetylation > 75%), HAuCl_4_, N-hydroxy- succinimide (NHS), nicotinamide adenine dinucleotide, 1-ethyl-3-(3-dimethylaminopropyl) carbodiimide (EDC), glutaraldehyde (Glu), K_4_Fe(CN)_6_, K_3_Fe(CN)_6_, thiamine pyrophosphate (TPP), and bovine serum albumin (BSA) were purchased from Sigma-Aldrich Chemical (Darmstadt, Germany). Phosphate buffer solution (containing NaCl, KCl, KH_2_PO_4_, Na_2_HPO_4_ 2H_2_O) was used. To adjust the pH values of the buffer solution, diluted NaOH or HCl solutions were used. All materials used were of analytical grade. For the preparation of required aqueous solutions, double-distilled water (DDW) was used.

All electrochemical measurements were performed with an Ivium potentiostat/galvanostat (Vertex, Ivium Technologies, Eindhoven, The Netherlands). In order to evaluate the obtained experimental data, the Ivium software package was used. A DRP-C110 SPCE (DropSens S.L., Llanera, Spain) including a carbon working electrode (3 mm in diameter), carbon counterelectrode, and a silver pseudo- reference electrode was provided. Cyclic voltammetry (CV), and electrochemical impedance spectroscopy (EIS) measurements were performed in the presence of a 1:1 mixture of 5 mM K_3_Fe(CN)_6_/K_4_Fe(CN)_6_ as a suitable redox-probe in 0.1 M PBS (pH = 7.4), using an alternating current voltage of 10 mV and frequency range of 0.1 Hz to 100 kHz. The CVs were recorded in −0.3 V to 0.7 V with a scan rate of 0.1 V/s. FTIR spectra were obtained on a model Spectrum GX spectrophotometer (Perkin-Elmer, Norwalk, CT, USA). The morphology and structure of the as-synthesized nanomaterials were examined by transmission electronic microscopy (TEM) (LEO 912 Omega, Zeiss, Germany) and scanning electron microscopy (SEM, Electro Scan 2020 Philips, Amsterdam, The Netherlands). XRD patterns were obtained on by 38066 Riva, d/G. Via M. Misone, 11/D (TN), Italy.

### 2.2. Preparation of the Antibody and Antigen

Toxin production, purification and detoxification and polyclonal antibody production and purification against botulinum neurotoxin methods were reported in our previous work [15].

### 2.3. Preparation of CSNPs

CSNPs were prepared according to a method reported by Koukaras and coworkers [24]. Briefly, nanoparticles were obtained upon the addition of an aqueous solution of TPP to a solution of CS in acetic acid (pH 3.5) with final concentrations of 0.5 and 2 mg·mL^−1^, respectively. The formation of nanoparticles was a result of the interactions between the negative groups of TPP and the positively charged amino groups of CS.

### 2.4. Preparation of Proposed Immunosensor

**Scheme 1:**
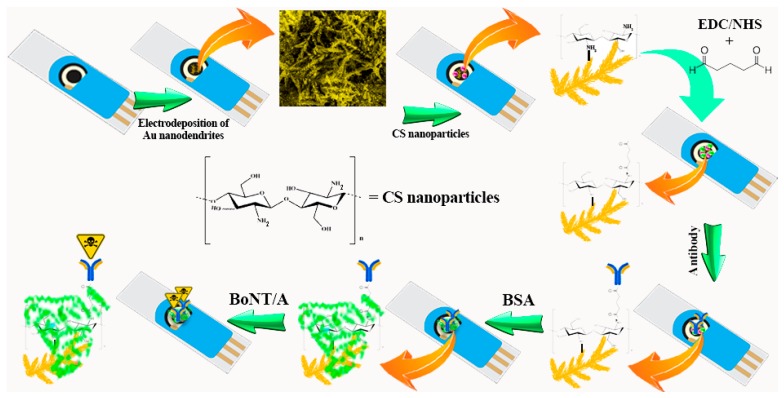
Fabrication process of the immunosensor.

AuNDs were synthesized electrochemically on the working electrode surface of the SPCE by a square-wave voltammetry technique. A solution containing 2 mM HAuCl_4_ and 150 mM nicotinamide adenine dinucleotide (NAD+) in 0.5 M H_2_SO_4_ was used as the electrolyte. The deposition of AuNDs was achieved using lower and higher potentials of −0.8 and 0.2 V (frequency of 40 Hz for a period of 2000 s), respectively. The electrode was then washed with copious amount of DDW and used in further experiments [25]. Then, AuNDs/SPCE was immersed in synthesized CSNPs solution for 10 h in 4 °C. CSNPs were attached to the electrode surface by forming the self-assembled monolayers (SAMs) between the amino groups of CS with electrodeposited AuNDs. To remove any unbound nanoparticles, the modified electrode was washed with DDW. Afterward, the modified SPCE was placed at a home-made electrochemical cell. The activated Glu solution is prepared by mixing of its solution with 20 mM EDC and 10 mM NHS solutions for 2 h at room temperature. Then, the activated Glu was dropped on the surface of the electrode. To avoid evaporation, the electrochemical cell was covered with Parafilm and held at room temperature. After 2 h, the electrode surface was rinsed with DDW. After this step, 5 µL of 100 µg·mL^−1^ antibody in PBS 7.4 was dropped on the modified working electrode surface of the SPCE, and the electrochemical cell was covered and incubated for 1 h at 4 °C. The electrode surface was then rinsed with PBS to remove any unbound antibody. Finally, the working electrode surface was blocked by BSA solution (0.05% w/v) for 1 h in 4 °C. BSA blocks any remaining active sites on the electrode surface. The prepared immunosensor was kept in a refrigerator (4 °C) before usage. The immunosensor fabrication steps are shown in Scheme 1.

### 2.5. Preparation of Real Sample

Milk and serum samples were used without sample filtration or other preparation steps [5,26]. Same volumes (10 μL) of diluted toxin (100, 1000 and 10,000 pg·mL^−1^) were spiked into 990 μL of all liquid matrices. The BoNT/A final concentrations in samples were 1.0, 10 and 100 pg·mL^−1^. Then all samples were analyzed with the proposed immunosensor.

## 3. Results

### 3.1. Characterization of Synthesized CS Nanocomposite

**Figure 1:**
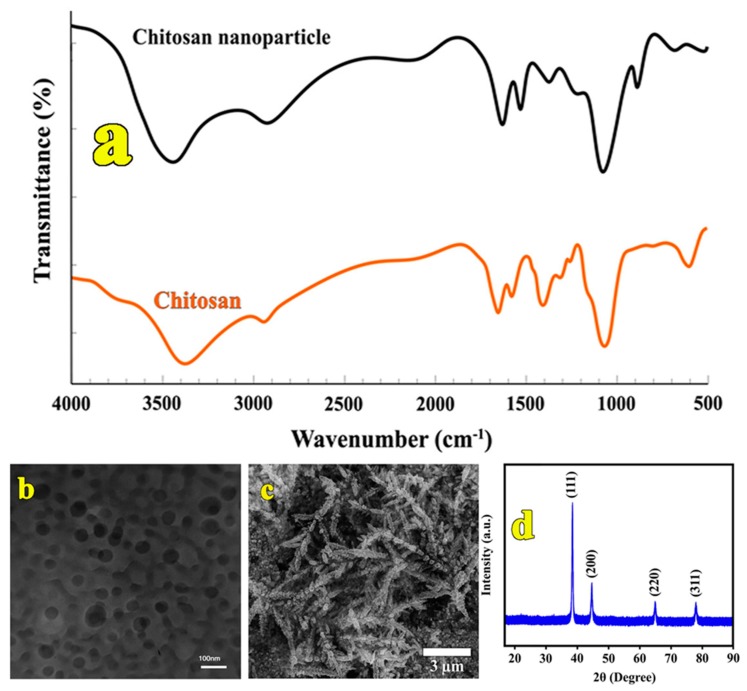
(**a**) FTIR spectra and; (**b**) TEM image of CNNPs; (**c**) SEM image and; (**d**) XRD pattern of AuNDs.

The synthesized CSNPs were characterized by the FTIR technique. The obtained FTIR spectra of CS and CSNPs are shown in Figure 1a. As can be seen, peaks at 3421, 1650 and 1591 cm^−1^ in the CS spectrum related to the stretching vibrations of amine and hydroxyl groups, the CONH_2_ group and bending vibrations of the NH_2_ groups are seen. A shift from 3421 to 3428 cm^−1^ was observed in CSNPs while the peak became wider compared to CS. These changes indicated that hydrogen bonding in nanoparticles increases compared with CS [27]. Also, the appearance of a new peak in 1630 cm^−1^ and a shift of the NH_2_ bending vibration peak from 1591 to 1542 cm^−1^ shows that the ammonium groups of CS interacted with the polyphosphoric groups of TPP. Therefore, by converting CS to CSNPs, both intra- and intermolecular interactions were enhanced [27,28,29].

In addition, the typical morphology and size of synthesized CSNPs were evaluated by TEM (Figure 1b). It was observed that CSNP are spherical in shape with the average diameters of 39 nm, indicating that CSNPs had been successfully synthesized [30,31,32]. Also, a SEM image of the electrosynthesized AuNDs on the electrode surface is shown in Figure 1c. As can be seen, AuNDs have well-defined uniform nanodendrite shapes where each nanodendrite has one long stem and several branches.

XRD analysis was performed to investigate the crystalline structure of the synthesized AuNDs (Figure 1d). The peaks at 38.3°, 44.4°, 64.6° and 77.6° related to the (1 1 1), (2 0 0), (2 2 0) and (3 1 1) planes are in good agreement with a reference face-centered cubic (FCC) Au pattern, respectively. These results confirmed that the AuNDs formed well-crystalized structures [20,25,33].

### 3.2. Characterization of the Immunosensor (CV and EIS)

The Ivium software was used for fitting the obtained impedance spectra and calculation of the values of the Randles (equivalent circuit) parameters involving the uncompensated resistance of the electrolyte (R_s_), capacitance of the dielectric layer (C_dl_), Warburg impedance (Z_w_, accounts for the diffusion of ions from bulk electrolyte to the electrode interface) and *R_CT_* [34].

**Figure 2:**
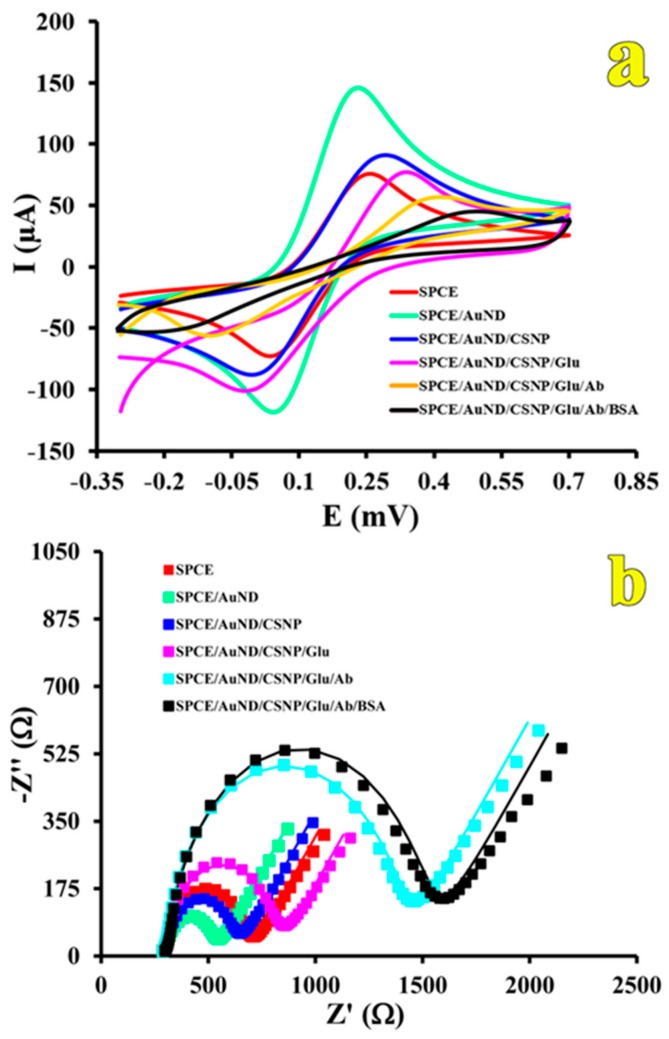
(**a**) CV and (**b**) EIS responses for step by step fabrication of proposed immunosensor. Measurements were performed in PBS (0.1 M and pH= 7.4), containing 5 mM of [Fe(CN)_6_]^3−/4−^ and 0.1 M KCl.

The obtained voltammograms and Nyquist plots (dot plots: experimental and line plots: fitted spectrum) from the step by step fabrication of the immunosensor are shown in Figure 2. As illustrated, after electrodeposition of AuNDs on the SPCE electrode surface, the peak separation decreased and the peak current of the electrochemical probe increased in comparison with the bare SPCE. The *R_CT_* was also dramatically decreased. These changes indicate that AuNDs can improve the electron transfer due to the increase in electrode surface area and conductivity. After surface modification by CSNPs, a decrease in oxidation peak current and increase in peak separation and *R_CT_* was observed. In the next step, with a coating of Glu on the electrode surface, voltammograms showed an increase in peak separation and a decrease in peak current (in Nyquist plots, *R_CT_* increased). These results can be due to its low electrical conductivity [35]. Incubation of the electrode with antibody led to an increase the diameter of the semicircle in the Nyquist plot and peak separation in the voltammograms with a decrease in peak current. Eventually, in order to block the probably remaining free spaces on the electrode surface, BSA was immobilized on the electrode surface and its hindrance on the electronic conductivity of modified electrode was observed. These results suggested that insulating protein and CSNPs layers on the electrode surface hinder the electron transfer between the redox probe and electrode. The values related to peak separations and peak currents in CVs and Randles, parameters in the fitting of electrochemical impedance spectra are presented in Table 1.

### 3.3. Optimization of the Antibody Immobilization Conditions

We used a technique of immobilization of antibody onto the electrode surface based on covalent bond formation between the carboxylic acid groups of Glu (on the electrode surface) and the amine groups in the FC fragment of the antibody. Glu is a bis-aldehyde species with two reactive ends. Glu can therefore crosslink two amine functional groups, for example, between two proteins or between a protein and a surface-immobilized species with amine groups (e.g., CSNPs). Carboxylates (–COOH) may react with NHS in the presence of EDC, resulting in a semi-stable NHS ester, which may then be reacted with primary amines (–NH_2_) to form amide crosslinks.

The key factors to obtain a sensitive response from the fabricated immunosensor are the concentration and incubation time of the immobilized antibody. Thus, in order to obtain the best immunosensor performance, these parameters were optimized. The results showed that by increasing the antibody concentration to 90 µg·mL^−1^, the *R_CT_* increased and stabilized at higher concentrations. This indicated that the electrode surface was saturated with the antibody and a further increase in the concentration of antibody will not lead to any further increase in R*_CT_*. Therefore, 100 µg·mL^−1^ was selected as an optimum antibody concentration for the fabrication of the immunosensor. *R_CT_* also increased with incubation time until it reached 55 min. A further increase in incubation time resulted in no further increase in *R_CT_*. As a result, 60 min was selected as the optimal time for antibody immobilization in this work.

### 3.4. Optimization of Assay Conditions

The electrochemical performance of the immunosensor would be also influenced by other factors, such as pH, temperature and incubation time. In order to evaluate the effects of these parameters on the immunosensor response, the relative change in the *R_CT_* (%∆*R_CT_*) is calculated by the following equation [36]:

(1) $$\%\Delta R_{CT}=\frac{R_{CT\left(BoNT/A\right)}-R_{CT\left(BSA\right)}}{R_{CT\left(BSA\right)}}\;\times 100$$

where *R_CT_*_(BoNT/A)_ is the value of the *R_CT_* after BoNT/A coupling with the immobilized anti-BoNT/A on the immunosensor. *R_CT_*_(BSA)_ also represents the value of the *R_CT_* after blocking the remaining sites on the electrode surface by BSA.

**Figure 3:**
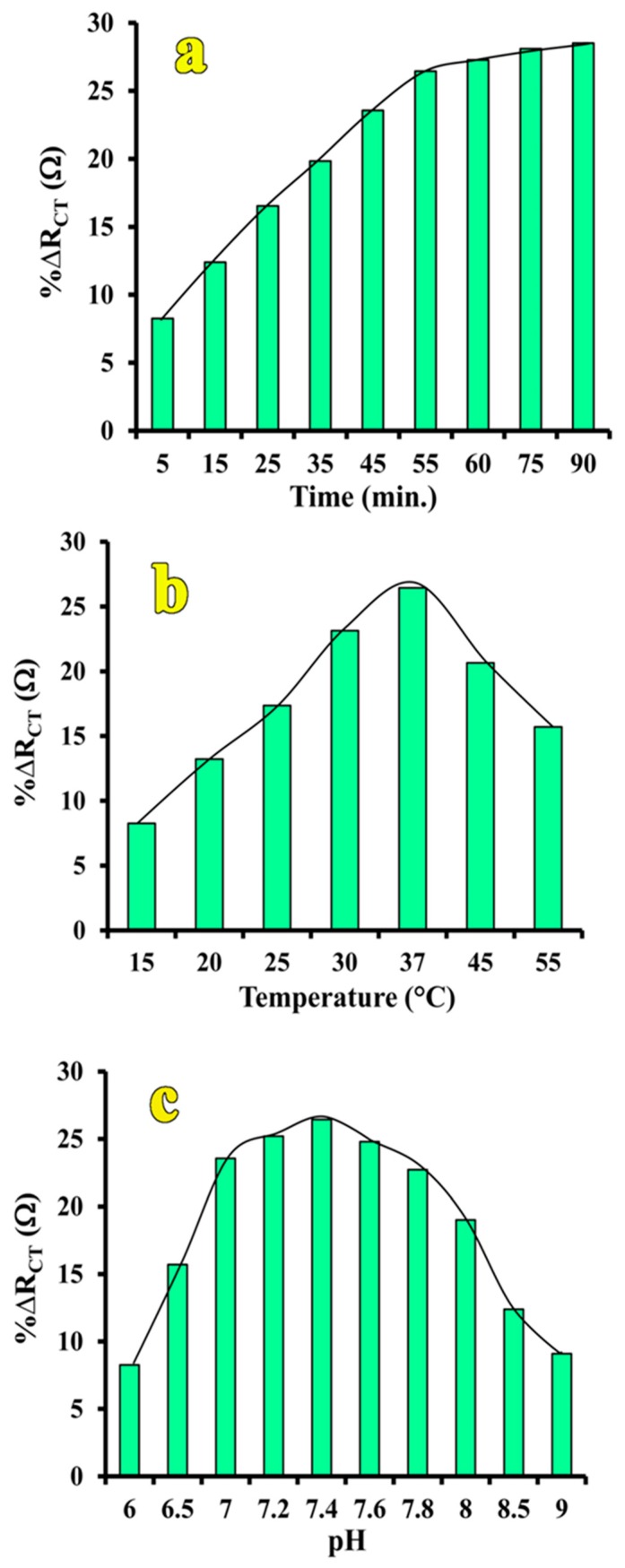
Optimization of the immunoreaction conditions: (**a**) time; (**b**) temperature and (**c**) pH.

After incubation of the immunosensor with 1 pg·mL^−1^ of BoNT/A during 5 to 60 min, %∆*R_CT_* was dramatically increased and then reached equilibrium (Figure 3a). The formed immunocomplex between the antibody and BoNT/A reached to equilibrium in this time and then no significant change with an increase in time occured. Thus, 60 min was selected as optimal immunoreaction time.

To optimize the immunoreaction temperature, the temperature was changed from 15 to 55 °C. As can be seen in Figure 3b, %∆*R_CT_* increased with the increase in temperature to 37 °C and then started to decrease. This suggested that the formation of immunocomplex at higher temperatures decreases in comparison with physiological temperature, related to a possible denaturation of the BoNT/A protein with increasing temperature. Therefore, 37 °C was selected as the optimum temperature for subsequent studies.

The effect of the pH on the immunosensor response was also studied in the range of 6.0 to 9.0. The immunosensor response increased with increasing pH value and the maximum relative change in the %∆*R_CT_* was obtained at pH 7.4 (Figure 3c). Thereafter, with an increase in pH, %∆*R_CT_* decreased, indicating that a highly alkaline environment would damage the stability and activity of antibody and immobilized proteins [15].

### 3.5. Analytical Performance of the Immunosensor

**Figure 4:**
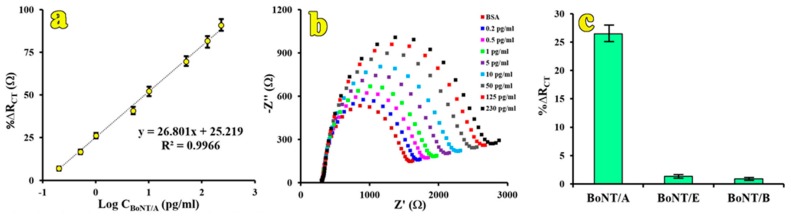
(**a**) Impedimetric immunosensor Nyquist plots and (**b**) Calibration curve (%∆*R_CT_* vs. log C) for BoNT/A different concentration. (**c**) The performance of immunosensor in E and B serotype of BoNTs. (C_BoNT/A_ = 1 pg·mL^−1^ and C_BoNT/E/B_ = 100 pg·mL^−1^).

Under the optimized conditions, the analytical performance of explained immunosensor was investigated using the reaction of the immunosensor with different BoNT/A concentrations. It was seen that the relative change in the *R_CT_* were proportional to BoNT/A concentrations in a range from 0.2 to 230 pg·mL^−1^. The linear regression equation was obtained as %∆*R_CT_* (Ω) = 26.801 log C_(BoNT/A)_/pg·mL^−1^ + 25.219 (R^2^ = 0.9966). The detection limit for proposed immunosensor was calculated to be 0.15 pg·mL^−1^ (based on signal/noise ratio of 3). The Nyquist plots and a calibration curve for BoNT/A determination are shown in Figure 4a,b, respectively.

Three modified electrodes independently prepared under the same condition, were evaluated next in reproducibility tests. A relative standard deviation (RSD%) of 2.3% was obtained for the oxidation current of 1 pg·mL^−1^ of BoNT/A, indicating high reproducibility of the fabrication method.

Also, the storage stability of fabricated immunosensor was investigated. Six immunosensors were fabricated and kept in a 0.1 M PBS pH = 7.4 containing 0.02% sodium azide, in a refrigerator at 4 °C. Then, the impedimetric response of each immunosensor toward the 1 pg·mL^−1^ of BoNT/A was recorded in a day. The results indicated that 98.7% and 90.3% of initial immunosensor response remained after 1 and 4 days, respectively. After 5 days of storage period had passed, just 81.1% of the initial response remained. Therefore, 4 days was considered as the maximum immunosensor stability.

It has been concluded that BoNT/A, BoNT/B, and BoNT/E are the species most frequently associated with human botulinum poisoning [37]. Therefore, cross-reactivity of the explained immunosensor for BoNT/A toward these serotypes was investigated in this study (Figure 4c). To achieve this goal, we applied the affinity purification strategy for amplifying the selectivity of the prepared polyclonal antibodies [15]. After using cross-absorbed polyclonal antibodies, the concentration of BoNT/A was detected at 1 pg·mL^−1^ while the concentration of the two other serotypes was 100 pg·mL^−1^. The obtained results indicated that no cross-reactivity is observed and the proposed immunosensor has good selectivity towards the B and E BoNTs serotypes.

### 3.6. Application of the Immunosensor in Real Samples

In order to evaluate the analytical performance of the proposed immunosensor, serum and milk samples were chosen as real samples and analyzed by the explained method. The obtained results are shown in Table 2. As can be seen, satisfactory RSDs and recoveries from the analysis of these samples were obtained. The obtained results were similar, with a relative error of less than 5% in milk and serum samples. These errors for 1.0 pg·mL^−1^ BoNT/A in milk and serum compared to the equivalent amount in buffer (Δ*R_CT_* = 664 Ω) were 4.49% and 4.33%, respectively. Also, the relative errors for 10.0 pg·mL^−1^ for BoNT/A determination were obtained to be 4.06% and 3.8% for milk and serum samples, respectively. Thus, it can be considered that the matrix of the milk and serum samples does not make a significant interference in the determination of BoNT/A by the proposed immunosensor. The obtained results were also, in good agreement with results obtained by a standard ELISA test.

## 4. Conclusions

A label-free impedimetric immunosensor based on the AuNDs/CSNPs composite for the direct detection of BoNT/A was manufactured. Its preparation consists of successive modification steps of a SPCE: (I) modification of the activated SPCE surface with AuNDs; (II) formation of a CSNPs self-assembled monolayer on the AuNDs; (III) dropping a Glu solution activated by EDC/NHS on the surface of the modified SPCE; (IV) covalent immobilization of BoNT/A polyclonal antibody; (V) covering any remaining active sites on the electrode with BSA. The new immunosensor combines the unique and attractive electronic behavior of AuNDs, the high specific surface area of CSNPs with an excellent specificity of BoNT/A antibody. The resulting nanocomposite was characterized by various characterization methods, including FTIR, TEM, SEM, XRD, CV and EIS techniques.

Under optimized condition, the %Δ*R_CT_* of the biosensor was proportional to BoNT/A concentration over the range of 0.2–230 pg·mL^−1^ with a detection limit of 0.15 pg·mL^−1^. Therefore, the immunosensor exhibited excellent reproducibility, stability and applicability for the practical use in comparison with many previously reported methods (Table 3).

## Figures and Tables

**Table 1:** Randles’ and CV parameters for step by step fabrication of proposed immunosensor related to Figure 2.

Electrode	I_pa_ (µA)	∆E_p_ (mV)	*R_CT_* (Ω)	R_s_ (Ω)	W (Mho)	Q	
						Y0 (µMho)	N
SPCE	74.4	210	401	291	2.39	2.81	0.883
SPCE/AuNDs	138	180	244	289	1.76	3.35	0.858
SPCE/AuNDs/CSNPs	84.5	271	339	290	1.66	2.80	0.882
SPCE/AuNDs/CSNPs/Glu	76.85	322	526	293	1.41	2.20	0.914
SPCE/AuNDs/CSNPs/Glu/Ab	43.9	384	1100	294	1.48	2.07	0.922
SPCE/AuNDs/CSNPs/Glu/Ab/BSA	27.8	428	1210	304	1.56	2.24	0.914

**Table 2:** The obtained immunosensor performance results in real samples.

Samples	Added (pg·mL^−1^)	Found (pg·mL^−1^)	Recovery (%)	RSD (%) (N = 3)	ELISA
Serum	0.00	-	-	-	N. D. *
	1.00	0.984	98.4	4.33	N. D.
	10.0	10.31	103.1	3.8	N. D.
	100.0	97.4	97.4	2.32	102.9
Milk	0.00	-	-	-	N. D.
	1.00	1.022	102.2	4.49	N. D.
	10.0	9.88	98.8	4.06	N. D.
	100.0	101.9	101.9	2.53	102.2

* Not Detected.

**Table 3:** Comparison of different methods for the determination of BoNT/A.

Detection Method	Dynamic Range	Analysis Time	Detection Limit	Ref.
Fluorescence	15–800 ng·mL^−1^	20 h	10 ng·mL^−1^	[7]
Fluorescence	20–300 pg·mL^−1^	4 h	21.3 fg·mL^−1^	[8]
Au nanoparticles/graphene-chitosan/EIS ^1^	0.27–268 pg·mL^−1^	60 min	0.11 pg·mL^−1^	[15]
EIS	25–125 fg·mL^−1^	30 min	25 fg·mL^−1^	[23]
CV ^2^	4–35 pg·mL^−1^	-	1 pg·mL^−1^	[38]
LSV ^3^	10 pg·mL^−1^–10 ng· mL^−1^	65 min	5 pg·mL^−1^	[39]
DPV ^4^	1 pg·mL^−1^–1 ng·mL^−1^	-	1 pg·mL^−1^	[40]
Enzyme linked Immunosorbent assay	-	5 h	163 pg·mL^−1^	[41]
Immunochromatographic method	-	15–30 min	5 ng·mL^−1^	[42]
Mass spectrometry	-	-	50 ng·mL^−1^	[43]
Gold nanodendrites/chitosan/EIS	0.2–230 pg·mL^−1^	60 min	0.15 pg·mL^−1^	This work

^1^ Electrochemical impedance spectroscopy, ^2^ Cyclic voltammetry, ^3^ Linear sweep voltammetry, ^4^ Differential pulse voltammetry.
